# Warty Carcinoma Penis: An Uncommon Variant

**DOI:** 10.1155/2017/2937592

**Published:** 2017-01-05

**Authors:** Sushma Thapa, Arnab Ghosh, Santosh Shrestha, Dilasma Ghartimagar, Raghavan Narasimhan, OP Talwar

**Affiliations:** ^1^Department of Pathology, Manipal College of Medical Sciences, Pokhara, Nepal; ^2^Department of Surgery, Western Regional Hospital, Pokhara, Nepal

## Abstract

Penile carcinoma frequency varies widely in different parts of the world and comprises 1–10% of all the malignancies in males. Majority of the cases of penile carcinoma are squamous cell carcinoma of penis comprising 60% to 70% of all cases. Warty carcinoma of penis is an unusual neoplasm and a variant of penile squamous cell carcinoma comprising 5%–10% of all the variants. The other histological variants include basaloid, verrucous, papillary, sarcomatous, mixed, and adenosquamous carcinoma. The various histological entities with an exophytic papillary lesions including warty carcinoma are together referred to as the “verruciform” group of neoplasms. The warty carcinoma has to be differentiated from these lesions and is typically distinguished by histological features of hyperkeratosis, arborescent papillomatosis, acanthosis, and prominent koilocytosis with nuclear pleomorphism. We present a case of 65-year-old male with growth measuring 6 × 4 cm in the penis who underwent total penectomy and was diagnosed as warty carcinoma penis.

## 1. Introduction

Penile carcinoma frequency varies widely in different parts of the world and comprises 1–10% of all the malignancies in males [1]. Warty carcinoma (WC) of the penis is an unusual neoplasm and a variant of penile squamous cell (SC) carcinoma [2]. It comprises 5%–10% of all the penile carcinomas [3]. The diagnosis is typically made by histological features of hyperkeratosis, arborescent papillomatosis, acanthosis, and prominent koilocytosis with nuclear pleomorphism [2].

## 2. Case Presentation

**Figure 1:**
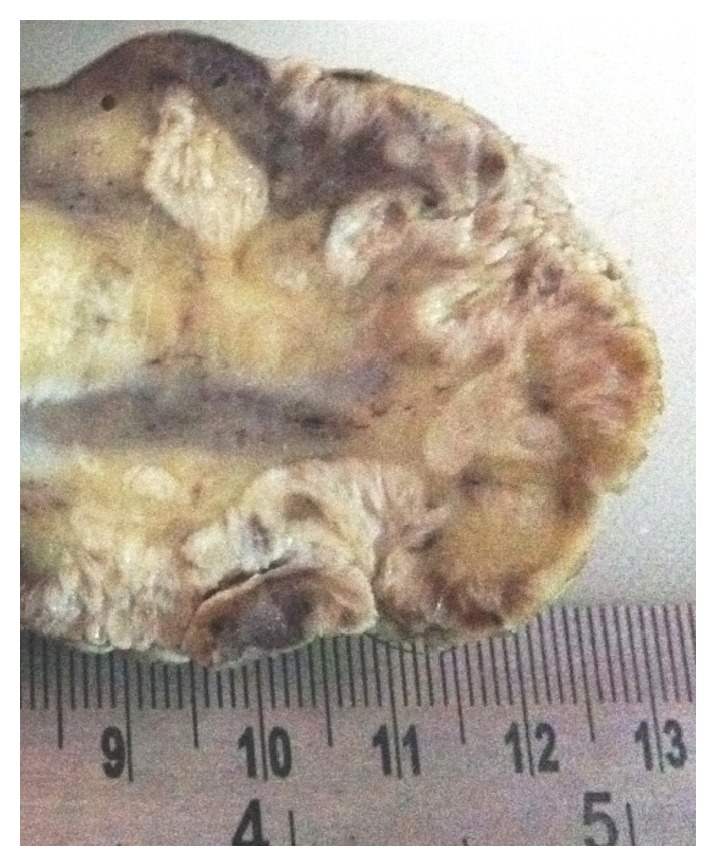
Close-up view of total penectomy specimen showing papillomatous growth.

**Figure 2:**
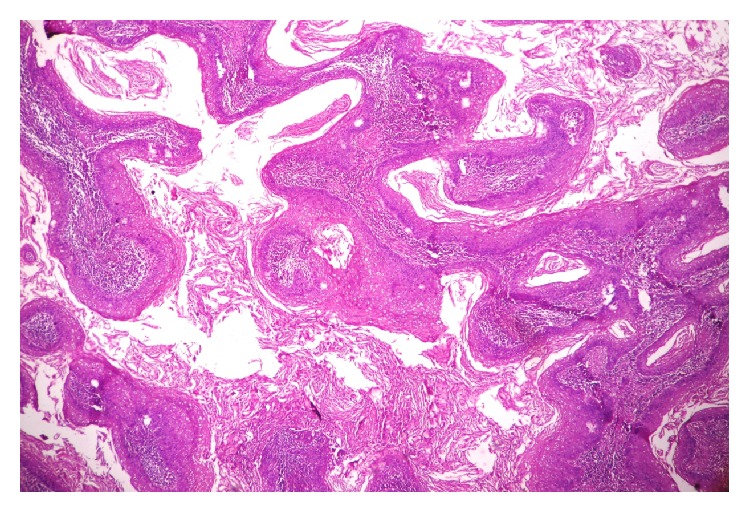
Arborizing papillomatosis with parakeratosis and acanthosis (H&E, ×100).

**Figure 3:**
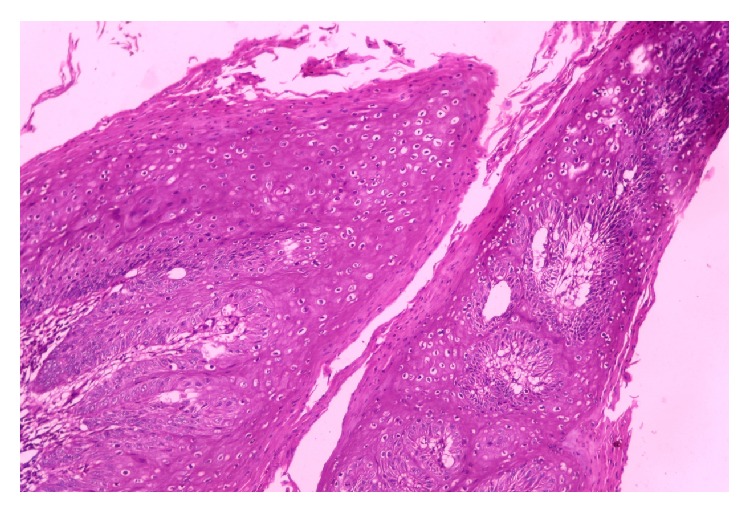
Conspicuous koilocytotic atypia present throughout the tumor (H&E, ×400).

A 65-year-old male presented with mass in the penis for 18-month duration. It was insidious in onset and gradually progressive. On general examination, the condition of the patient was fair. All the vitals were in stable conditions. There was no palpable inguinal lymphadenopathy. On local examination, there was a large fungating penile mass identified in the glans penis and extending to the shaft of penis. Total penectomy was done and sent for histopathological examination. Grossly, the specimen showed an exophytic, gray white firm mass involving the glans penis and coronal sulcus and extending up to the prepuce and shaft of penis measuring 6 × 4 cm. The cut surface showed a papillomatous growth (Figure 1). On microscopic examination, the lesion had an arborescent papillomatosis with prominent fibrovascular core (Figure 2). Parakeratosis, acanthosis, and koilocytosis with nuclear pleomorphism were present throughout (Figure 3). The case was reported as WC of the penis with uninvolved surgical margins. On follow-up after 1 year, patient was doing fine and there was no recurrence.

## 3. Discussion

Carcinoma of the penis is rare in developed countries and the frequency is high in Asia (China, Vietnam, Sri Lanka, Burma, and India), Africa (Uganda), and Latin America (Mexico) comprising 10% of all the malignancies [4]. The etiology is typically multifactorial and includes poor hygiene, lack of circumcision, preexisting condyloma acuminatum, squamous intraepithelial lesions with warty features, and HPV infections [4]. The majority of penile neoplasms are SC, but within this category a heterogeneous variety of growth patterns and histologic subtypes may be seen. The different histological variants include basaloid, verrucous, papillary, sarcomatous, mixed, adenosquamous, and warty carcinoma (WC) [2]. WC of the penis is an unusual variant of penile SC comprising only 5%–10% [4]. The first description of WC of the penis in English literature can be found in the article by Davies [5] in 1965 where it has been referred to as malignant condyloma. Similar carcinoma has been described much earlier in the vulva by Rastkar et al. [6] in 1982. The first series of 11 cases of penile WC was published in 2000 by Cubilla et al. [2]. Different authors further found an increased association of human papilloma virus (HPV) infection with vulval as well as penile WC [7–9]. Approximately 40% of penile cancers have been shown to be attributable to HPV types 16 and 18. Type 16 has preferentially been associated with a small subset of penile cancers, including basaloid, mixed warty-basaloid, and pure warty carcinomas [2, 10]. The average age of presentation is 5th decade [11]. Our case presented in 6th decade. In one case series, the patients from the United States were younger than those from Paraguay [2]. The average duration of the disease ranged from 2 to 60 months (average of 19 months) which is similar to our case.

Different histological groups of penile neoplasms with an exophytic papillary lesions including WC are collectively referred to as the “verruciform” group of neoplasms [2, 12]. Exophytic verruciform lesions of the penis include

- condyloma acuminatum;
- giant condyloma acuminatum;
- warty (condylomatous) squamous cell carcinoma;
- warty-basaloid carcinoma;
- verrucous carcinoma;
- papillary squamous cell carcinoma, not otherwise specified;
- carcinoma cuniculatum.

Histologically, a papillomatous pattern with acanthosis is noted in all the cases of verruciform lesions and thus they may mimic each other both clinically and on microscopy (Table 1) [2, 10–12]. WC is diagnosed when typical features, namely, papillomatous exophytic growth with rounded papillae, prominent fibrovascular cores, irregular infiltrative tumor interface, and conspicuous koilocytosis, are present. Fibrovascular cores are prominent in WC, papillary carcinoma, not otherwise specified (NOS), and giant condyloma and mostly absent in verrucous carcinoma. Koilocytosis is characterized with clear perinuclear cytoplasmic haloes, wrinkled, enlarged nuclei, bimultinucleated cells, and dyskeratosis [2]. Koilocytic atypia is characteristically seen throughout the tumor in WC and may be seen on the surface in giant condyloma and absent in verrucous and papillary carcinoma, NOS. In verrucous carcinoma, the papillae are regular and the base is characteristically broad and pushing [13]. In papillary carcinoma, NOS, the tip of the papillae is polymorphic, namely, straight, undulated, spiky, rounded, or blunt, and the interface of the tumor and stroma is irregular, but the prominent condylomatous papillae and conspicuous pleomorphic koilocytosis, hallmark of warty tumors, are absent [14]. Warty-basaloid carcinoma also needs to be differentiated from pure WC. It has got 3 histological patterns in which the most common type shows warty growth on the surface and basaloid features in deep infiltrative nest [10]. Carcinoma cuniculatum is characterized by exoendophytic growth with irregular and deep sinuses and tracts connecting the surface of the neoplasms to deep anatomic structures [11, 12].

MRI of the penis to identify invasion into the corpora cavernosa or spongiosum is helpful when the depth and extent of tumor remain unclear on physical examination [4]. Abdominal and pelvic CT or MRI may be useful to exclude metastatic disease. Partial penectomy with a 2 cm proximal resection margin remains the gold standard treatment [4]. But in our case total penectomy was done due to extension of the tumor into the proximal shaft of the penis. In a study done by Cubilla et al. [15], three prognostic groups of penile SC were identified in relation to different histologic subtypes and outcome. The verruciform tumors had a better prognosis compared to SC of the usual type which in turn had a better prognosis than the basaloid-sarcomatoid group. Guimarães et al. [16] have documented a 10% recurrence rate for WC when compared to 28% for usual SC. Metastatic rate of papillary carcinoma, NOS, is similar to WC and higher than verrucous carcinomas but lower than usual SCs [14]. The incidence of metastasis in warty-basaloid tumors is similar to that of usual SC, higher than WC, and lower than basaloid carcinomas [10].

## 4. Conclusion

Warty carcinoma of penis is an unusual variant of squamous cell carcinoma. The various histological entities with exophytic papillary lesions together known as “verruciform” group of neoplasms of penis should be considered as differential before a definite diagnosis.

## Figures and Tables

**Table 1:** Comparison of warty carcinoma and other verruciform lesions.

	Giant condyloma	Warty carcinoma	Verrucous carcinoma	Warty-basaloid carcinoma	Carcinoma cuniculatum	Papillary carcinoma, NOS
Average size	>8 cm	>5 cm	<3.5 cm	2–12 cm	5–8.9 cm	3–14 cm
Papillae	Arborizing, nonundulating, rounded	Long and undulating, Arborizing, rounded, or tapered	Straight	Similar to warty carcinoma	Straight or irregular	Irregular, complex
Fibrovascular cores	Prominent	Prominent	Rare	Present in papillomatous variant	Present	Present
Koilocytotic atypia	Present at surface	Prominent and diffuse	Absent	Present on the surface	Absent	Absent
Base	Regular, broad, and pushing	Rounded or irregular and jagged	Regular, broad, and pushing	Rounded or irregular and jagged	Rounded or irregular and jagged	Irregular and jagged
HPV	Type 6–12	Type 16	Absent	Type 16	Absent	Absent
